# Microbial Community Structure and Ecological Networks during Simulation of Diatom Sinking

**DOI:** 10.3390/microorganisms10030639

**Published:** 2022-03-17

**Authors:** Ying Liu, Mengchu Zeng, Zhe Xie, Daliang Ning, Jizhong Zhou, Xi Yu, Rulong Liu, Li Zhang, Jiasong Fang

**Affiliations:** 1 Hadal Science and Technology Research Center, Shanghai Ocean University, Shanghai 201306, China; liuying06shengke@163.com (Y.L.); xiezhe06@163.com (Z.X.); yuxihb@126.com (X.Y.); rlliu@shou.edu.cn (R.L.); 2 State Key Laboratory of Geological Process and Mineral Resources, Faculty of Earth Sciences, China University of Geosciences, Wuhan 430074, China; ZMengchu@163.com; 3 Department of Microbiology and Plant Biology, Institute for Environmental Genomics, University of Oklahoma, Norman, OK 73019, USA; ningdaliang@ou.edu (D.N.); jzhou@ou.edu (J.Z.); 4 School of Civil Engineering and Environmental Sciences, University of Oklahoma, Norman, OK 73019, USA; 5 Earth and Environmental Sciences, Lawrence Berkeley National Laboratory, Berkeley, CA 94704, USA; 6 State Key Joint Laboratory of Environment Simulation and Pollution Control, School of Environment, Tsinghua University, Beijing 100084, China; 7 Laboratory for Marine Biology and Biotechnology, Qingdao National Laboratory for Marine Science and Technology, Qingdao 266237, China

**Keywords:** hydrostatic pressure, particle-attached microorganisms, free-living microorganisms, particulate organic matter, DNA-SIP, microbial interactions

## Abstract

Microbial-mediated utilization of particulate organic matter (POM) during its downward transport from the surface to the deep ocean constitutes a critical component of the global ocean carbon cycle. However, it remains unclear as to how high hydrostatic pressure (HHP) and low temperature (LT) with the sinking particles affects community structure and network interactions of the particle-attached microorganisms (PAM) and those free-living microorganisms (FLM) in the surrounding water. In this study, we investigated microbial succession and network interactions in experiments simulating POM sinking in the ocean. Diatom-derived ^13^C- and ^12^C-labeled POM were used to incubate surface water microbial communities from the East China Sea (ECS) under pressure (temperature) of 0.1 (25 °C), 20 (4 °C), and 40 (4 °C) MPa (megapascal). Our results show that the diversity and species richness of the PAM and FLM communities decreased significantly with HHP and LT. Microbial community analysis indicated an increase in the relative abundance of Bacteroidetes at high pressure (40 MPa), mostly at the expense of Gammaproteobacteria, Alphaproteobacteria, and Gracilibacteria at atmospheric pressure. Hydrostatic pressure and temperature affected lifestyle preferences between particle-attached (PA) and free-living (FL) microbes. Ecological network analysis showed that HHP and LT enhanced microbial network interactions and resulted in higher vulnerability to networks of the PAM communities and more resilience of those of the FLM communities. Most interestingly, the PAM communities occupied most of the module hubs of the networks, whereas the FLM communities mainly served as connectors of the modules, suggesting their different ecological roles of the two groups of microbes. These results provided novel insights into how HHP and LT affected microbial community dynamics, ecological networks during POM sinking, and the implications for carbon cycling in the ocean.

## 1. Introduction

Marine organic matter (OM) produced mostly by phytoplankton in the photic zone, and about 1–40% OM is transported vertically from the surface ocean to the deep ocean, largely through sinking of particulate organic matter (POM) [1,2,3,4,5], with a crucial role in shaping the structure, metabolic functions, and distributions of microbial communities in the deep ocean [5]. During the descent, the sinking POM, serving as nutrient-rich hotspots, is preferentially colonized by particle-attached microorganisms (PAM) that secrete extracellular enzymes to convert POM to dissolved organic matter (DOM), fueling the free-living microorganisms (FLM) in the surrounding waters [1,4,6]. Therefore, the fate of sinking POM in the deep ocean is largely dependent on the taxonomic composition, trophic lifestyles, and network interactions of the PAM and FLM communities in the whole water column [2,3,4].

Previous comparative investigations on the physiology, taxonomic composition, metabolic activity, and genomic characteristics of the PAM and FLM have greatly improved our understanding of the distinct ecological functions of PAM and FLM in the various marine environments [7,8,9,10,11]. For example, the cell size and abundance of PAM were relatively higher than that of FLM [3,8,12], and higher extracellular enzyme activities (EEA) were observed in the PAM communities than in their FLM counterparts [7,13,14]. Generally, the PAM and FLM communities exhibited different taxonomic composition [10,15,16,17]. However, many phylogenetic similarities between the PAM and FLM communities were observed in some studies [18,19,20,21], suggesting that some microbes could switch their lifestyles depending on particles availability and varied environmental factors [10,17,22]. Metagenomic studies revealed that PAM generally possessed larger genomes with functional genes related to motility, adhesion, and antibiotic resistance for rapidly nutrient-rich particles acquisition, colonization, and decomposition, whereas FLM had small and streamlined genomes for better adaptations to oligotrophic environments and uptake of recalcitrant DOC [8,11,23,24]. In addition, contrasting network interactions were observed in the PAM and FLM communities in the surface waters or during phytoplankton blooms [25,26,27,28]. However, these studies only focus on the upper ocean, and it is largely unknown how the PAM and FLM communities respond to increasing hydrostatic pressure and low temperature (LT) during POM sinking [20,29,30].

High hydrostatic pressure (HHP) has profound effects on microbial physiology, growth, taxonomic composition, metabolism, and evolution [30,31,32,33,34]. The accumulating knowledge on microbial adaptation to HHP indicated that increasing pressure led to reduction in bacterial abundance and diversity [30,35,36], inhibition of bacterial enzyme activities [29], decrease in organic matter degradation rates [20,37], and shift in community composition [20,30,35,36]. In addition, HHP inhibited respiration activity of microbial communities associated with the diatom aggregates, reduced POC degradation, and further enhanced the POM export to the deep ocean, and improved the efficiency of the biological carbon pump (BCP) [38]. Despite that the previous studies have addressed the physiological responses of microbes to HHP, large knowledge gaps remain as to the effects of increasing pressure on microbial succession and network interactions of PAM and FLM communities associated with sinking POM in the ocean.

In the present study, by exposing the same surface water microbial communities to the same diatom detritus (so as to eliminate the factor of changing chemistry of POM with depth or pressure in the ocean) under different hydrostatic pressures and temperatures, and combining sensitive DNA-SIP and 16S rRNA gene high through-put sequencing technology, we investigated changes in community structure, trophic lifestyle, and network interactions of the PAM and FLM. We hypothesized that HHP and LT exerted profound effects on microbial community structure, network interactions, and lifestyle preferences (PA or FL). The goal of this study was to determine how HHP and LT affects the community successions, network interactions, and lifestyle preferences of PAM and FLM during POM sinking.

## 2. Materials and Methods

### 2.1. Seawater Sampling

Seawater samples were collected from a water depth of 15 m at the Eastern China Sea (ECS, 30°39′48″ N, 122°29′48″ E) in September 2018 (Figure S1). The corresponding geochemical parameters of the water samples are listed in Table S1.

Triplicate 3 L of the seawater was filtered sequentially through 3.0 and 0.22 μm pore-size polycarbonate membrane (47 mm; Merck Millipore Ltd., Burlington, MA, USA) to obtain PAM and FLM, respectively [16], and the filters were preserved at −80 °C.

### 2.2. DNA-SIP Experiments

Diatom species *Thalassiosira weissflogii* (strain CCMA-102) was selected for the ^13^C-labeled POM experiment. The incubation system of *T. weissflogii* contained 5 L sterile artificial seawater (ASW) [39], 250 mL *Thalassiosira weissflogii* solution (logarithmic phase), f/2 media (dilution ratio, 1000:1), and 1 g NaH^13^CO_3_ or NaHCO_3_ (filtered through a 0.22 μm filter before use) [40]. Growth of diatom was performed in an illumination incubator at 20 °C for 20–30 days, with a 12-hour:12-hour light/dark regime. After incubation, the diatom detritus was centrifuged, lyophilized, and autoclaved as previously described [20]. The ^13^C atom percentage (%) of the ^13^C- and ^12^C-labeled diatom detritus was calculated to be 71.7% and 1.3%, respectively, by using an isotope ratio mass spectrometer (Thermo Scientific, Wilmington, DE, USA).

The ECS surface-water microorganisms were incubated with diatom-derived POM at different hydrostatic pressures (temperatures) of 0.1 (25 °C), 20 (4 °C), and 40 (4 °C) MPa for 48–88 h in the dark. The incubation media consisted of 500 mL surface seawater, 0.15 g sterile ^13^C-labeled particulate organic matter or ^12^C-POM (control), and 167 mL (25% of the total volume) Fluorinert^TM^ (3M^TM^ Corp., Minneapolis, MN, USA). Fluorinert was saturated with oxygen for 8 h at 4 °C and then added to serve as a source of oxygen [20,41]. Two controls were prepared in a similar way: 500 mL surface seawater and 167 mL Fluorinert (unamended control); 500 mL surface seawater, 0.15 g ^12^C-control POM, 167 mL Fluorinert, and 300 μL 80 g/L HgCl_2_ (killed control). All components of each incubation system were placed in a sterile pouch bags (Kapak SealPak 503, 710 mL; Figure S2a), and were then sealed with a heat sealer. Pressure incubations were conducted in several cylindrical, stainless steel pressure vessels with inner dimensions of 86 mm in diameter and 380 mm in length (Feiyu Petrochemical Instrument Equipment Inc., Nantong, China; Figure S2b), with a maximum working pressure of 60 MPa. Pressure was applied using a hand-operated pump with a quick-fit connector to the pressure vessel (Feiyu Petrochemical Instrument Equipment Inc., Nantong, China; Figure S2c). Triplicate incubations were carried out for all treatments. Bacterial growth plateaued after 48 or 88 h.

### 2.3. DNA Extraction and SIP Ultracentrifugation

After incubation, triplicate 350 mL of the incubation solution from ^13^C-labeled or ^12^C-control treatments was filtered as described above, and the filters were stored at −80 °C for further molecular microbiological analysis.

The filters were first cut into pieces and then transferred to 2 mL sterilized centrifuge tubes. The total DNA was extracted with FastDNA Spin Kit for Soil (MP Biomedical, Solon, OH, USA) according to the manufacturer’s protocols. The obtained total DNA was dissolved in 100 μL DNase-free ddH_2_O. The total DNA concentration was measured by NANODROP 2000 (Thermo Scientific, Wilmington, DE, USA) and preserved at −80 °C until further analysis.

The DNA-SIP ultracentrifugation and gradient fractionation was conducted by following the method as previously described [20,42,43]. Briefly, approximately 2.0 μg of the total ^13^C- or ^12^C-labeled DNA of PAM and FLM after incubation was mixed well with 0.9 mL gradient buffer (pH 8.0; 100 mM Tris HCl; 100 mM KCl; 1.0 mM EDTA) and 4.9 mL CsCl stock solution (1.85 g mL^−1^, Sigma, Welwyn Garden City, UK) to achieve an average buoyant density of 1.725 g mL^−1^, and then added into Quick-Seal 5.1 mL ultracentrifuge (13 × 51 mm, Beckman Coulter) with no bubbles by using a 10 mL injection syringe. After being sealed and balanced, the ultracentrifuge tubes were loaded symmetrically into a NVT 65.2 vertical rotor (Beckman Coulter, Inc., Palo Alto, CA, USA) and centrifuged at 190,000 *g* at 20 °C for 44 h in the ultracentrifuge (Optima XPN-100, Beckman Coulter) [42].

After centrifugation, fractionation of density gradient was performed by replacing the gradient mixtures with DNase-free ddH_2_O from the bottom to the top of the ultracentrifuge tube using a PHD ULTRA syringe pump (Harvard Apparatus) [42]. A total of 15 DNA gradient fractions were obtained, with each fraction about 340 μL, and 20 μL of each fraction was pipetted for measuring refractive index by applying an AR200 digital hand-held refractometer (Reichert, Depew, NY, USA). The empirical conversion formula of refractive index to buoyant density of each DNA fraction was as follows: ρ = −75.9318 + 99.2031x −31.2551x^2^, where ρ represents buoyant density, and x represents refractive index.

The fractionated DNA was purified from CsCl solution by adding 550 μL polyethylene glycol 6000 (PEG) and precipitated at room temperature for two hours, and then centrifugated at 13,000× *g* for 30 min. The obtained DNA was further washed two times with 70% ethanol, and final dissolved in 30 μL DNase-free ddH_2_O.

### 2.4. Quantitative PCR

The qPCR amplification of the fractionated DNA was performed on a 7500 Real Time PCR System (Applied Biosystems, Waltham, MA, USA) and analyzed by using ABI 7500 software v2.3. The triplicate 20 μL mixture included 10 μL GoTaq^®^ qPCR Master Mix (Promega Corporation, Madison, WI, USA), 2 μL of each primer (2 μM), 4 μL the fractionated DNA, and 2 μL qPCR H_2_O. The qPCR primers for the bacterial 16S rRNA gene were Bac338F (5’-ACTCCTACGGGAGGCAGCAG-3′) and 518R (5′-ATTACCGCGGCTGCTGG-3′) [44]. The thermal cycle program consisted of initial denaturation (95 °C) for 10 min; 40 cycles of denaturation at 95 °C for 15 s, annealing at 60 °C for 1 min, and extension at 72 °C for 1 min. Cycle of threshold (Ct) was determined using auto-baseline and auto-threshold functions in ABI 7500 software. Melting curves were used to assess the specificity of amplification. The standard curve (Ct vs. gene copy numbers) was constructed with a series of 10-fold diluted plasmid DNA containing fragments of *Pseudoalteromonas* sp. 16S rRNA gene, and the sequence of this plasmid DNA is presented in the Supplementary File S1. The 16S rRNA copy number of plasmid DNA was calculated according to an online calculator (http://scienceprimer.com/copynumber-calculator-for-realtime-pcr, accessed on 8 September 2020). In addition, blanks were run with DNase-free ddH_2_O. The amplification efficiency ranged from 95% to 103%, and the R^2^ values ranged from 0.99 to 1.0. The bacterial copy number was obtained by the average of three parallel samples.

### 2.5. PCR Amplification of 16S rRNA Gene

The total and fractionated DNA samples were amplified using the barcoded primers 515F (5′-GTGYCAGCMGCCGCGGTAA-3′) and 806R (5′-GGACTACNVGGGTWTCTA AT-3′) [45], targeting the V4 region of 16S rRNA gene. The 25 μL PCR reactions contained 10 μL Premix Taq^TM^ (Takara, China), 0.5 μL of each primer (10 μM), 10 ng of DNA, and 13 μL DNase-free ddH_2_O. The PCR was performed on a thermocycler PCR system (GeneAmp 9700, Applied Biosystems) with the following program: 94 °C for 3 min; 35 cycles of 94 °C for 45 s, 50 °C for 60 s, and 72 °C for 90 s; 72 °C for 10 min. Negative and positive control were contained in each run. All PCR amplicons were visualized on 1% agarose gels and purified using EZNA Cycle Pure Kit (Omega Bio-Tek, Norcross, GA, USA), then quantified using Qubit 3.0 Fluorometer (Invitrogen, Carlsbad, CA, USA).

### 2.6. Illumina MiSeq Sequencing and Data Processing

The amplicon samples were pooled in equimolar amounts and paired-end sequenced (2 × 300) with an Illumina MiSeq platform (Illumina, San Diego, CA, USA) at the Majorbio Bio-Pharm Technology Co.Ltd. (Shanghai, China). The raw data were quality-filtered by Trimmomatic [46] and then assembled by FLASH [47]. The chimeric sequences were detected and removed by UPARSE (Version 7.1) [48], and operational taxonomic units (OTUs) were clustered with 97% similarity cutoff using UPARSE. The taxonomy of each OTU was analyzed by Ribosomal Database Project (RDP) Classifier [49] against the Silva 16S rRNA database (SSU138) [50], with a confidence threshold of 70%. These processes produced 10,475–68,060 sequences of 78 different samples in the OTU table, and the sequences were resampled to the minimum sequences (10,475) among all samples. Alpha-diversity indices (e.g., Shannon, Simpson, Chao1, Ace, and the Good’s coverage) were calculated using Mothur (Version 1.35.1) [51].

### 2.7. Statistical and Ecological Analyses

Non-metric multidimensional scaling (NMDS) and the analysis of similarity (ANOSIM) were performed using PRIMER v7 package [52]. Linear discriminant analysis (LDA, http://huttenhower.sph.harvard.edu/galaxy/root?tool_id=PICRUSt_normalize, accessed on 22 February 2022) was used to identify potential biomarkers at different taxonomy classification levels, with an LDA score threshold of 5.0. The Venn diagram was conducted using Venny 2.1.0 [53].

In this study, “odds ratio” was used to assess microbial preference for the PA or FL lifestyle. The formula for the odds ratio is as follows: odds ratio = log10 (relative abundance in PA fraction/relative abundance in FL fraction), where odds ratio > 0 represents the PA preference, while odds ratio < 0 represents the FL preference [10,54]. We defined active communities as those with relative OTU abundance ≥1%, i.e., the abundance of an OTU retrieved in the 13C-heavy DNA fraction minus the corresponding abundance in the 12C-heavy DNA fraction, after incubations and SIP fractionation (Figure S3) [20,55].

### 2.8. Ecological Network Analysis

The ecological networks were constructed based on Molecular Ecological Network Analyses Pipeline (MENAP, http://ieg4.rccc.ou.edu/mena/). Only taxa detected in more than six samples were used for network analysis. Pearson correlation coefficients (r) between any two OTUs were estimated to generate the association matrix. The random matrix theory (RMT) approach was used to determine the threshold of correlation coefficient to construct the network of non-random associations [56,57]. Network properties were calculated using MENAP as previously described [57]. Module separation and modularity calculation were conducted by greedy modularity optimization [58], and the within-module connectivity (Zi) and among-module connectivity (Pi) [59] values for all nodes were calculated. The networks were visualized in Cytoscape (version 3.8.2) [60].

The nodes in the networks were divided into four topological roles based on Zi and Pi values: network hubs (highly connected nodes within entire network, Zi ≥ 2.5, Pi ≥ 0.62), module hubs (highly connected nodes within modules, Zi ≥ 2.5, Pi < 0.62), connectors (nodes that connect modules, Zi < 2.5, Pi ≥ 0.62), and peripherals (nodes connected in modules with few outside connections, Zi < 2.5, Pi < 0.62) [56,57,61,62].

The network parameters [56,61] used in this study were explained as follows:

Scale-free: In a scale-free network, most nodes have few neighbors, while only few nodes have large number of connected neighbors.

Small-world: In such a network, the average distance between two nodes is short, showing that the nodes in a network are always closely related with each other.

Average connectivity (avgK): Connectivity refers to the number of nodes directly connected by a node. It is the most commonly used concept for describing the topological property of a node in a network. Higher average connectivity usually means a more complex network.

Average clustering coefficient (avgCC): Higher avgCC means a node is fully connected with its neighbors; a value close to 0 means hardly any connections with neighbors.

Average geodesic distance (GD): Geodesic distance is the shortest path between two nodes. A smaller average geodesic distance means all the nodes in the network are closer; therefore, the network is more complex.

Modules: Network modules can be considered as niches or microbial functional units.

Modularity: modularity is the degree that a network can be divided into communities or modules. For ecological networks, microbial species in a module could be considered to have a similar ecological niche. The value of modularity varies from 0 to 1. The higher modularity is, the more modules a network can be divided into and therefore the less complex a network is.

ZP-plot: We used ZP-plot to distinct the roles that each node play in the network by analyzing two parameters including Zi and Pi. The roles of nodes can be classified into four different categories, including network hubs, module hubs, connectors, and peripherals.

## 3. Results

### 3.1. Microbial Diversity

A total of 1033 OTUs were identified from 3,615,779 high-quality sequences from 78 samples, with 929 and 835 OTUs (731 shared OTUs) for communities of PAM and FLM, respectively (Table S2). The Good’s coverage ranged from 99.8% to 100% (Table S3), suggesting that the diversities of the microbial communities were well covered in this study.

**Figure 1:**
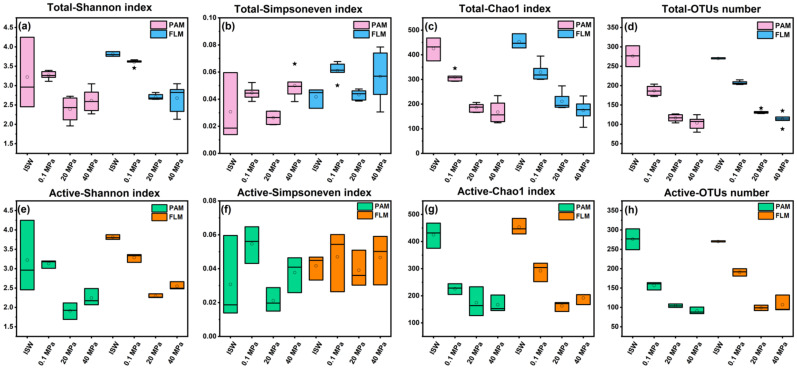
Alpha diversity indices (Shannon, Simpsoneven, Chao1) and OTUs number of the total and active PAM and FLM communities during POM sinking at ISW, 0.1, 20, and 40 MPa, respectively. (**a**) Total Shannon index; (**b**) total Simpsoneven index; (**c**) total Chao1 index; (**d**) total OTUs number; (**e**) active Shannon index; (**f**) active Simpsoneven index; (**g**) active Chao1 index; (**h**) active OTUs number. ISW represents in situ surface waters, hollow circle represents mean value, * represents outlier, and solid line represents median.

Overall, microbial alpha diversity indices (Shannon, Simpsoneven, and Chao1) and OTUs number of the total microbial communities showed a significantly decreasing trend with growth pressure (*t*-test, *p* < 0.05, Figure 1a–d and Table S4). On the other hand, the total FLM communities had significantly higher alpha diversity than the PAM communities at 0.1 and 20 MPa (*p* < 0.05, Figure 1a–d and Table S4). However, no significant difference was observed in alpha diversity between the two groups at the ISW (ECS in situ surface water) and 40 MPa (*p* > 0.05, Figure 1a–d and Table S4).

Similarly, microbial diversity of the active PAM and FLM communities also decreased significantly with increasing pressure (*p* < 0.05, Figure 1e–h and Table S4). Nevertheless, no significant differences were detected in microbial alpha diversity between the active PAM and FLM communities (*p* > 0.05, Figure 1e–h and Table S4).

**Figure 2:**
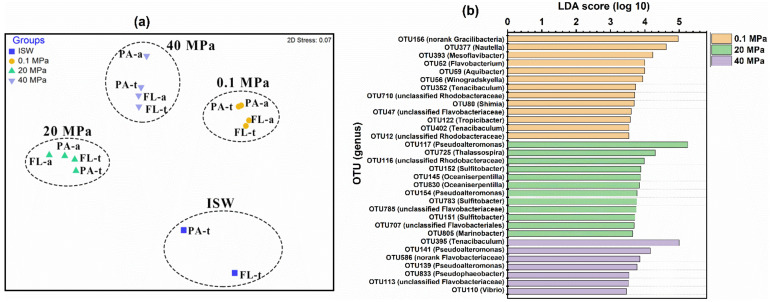
Nonmetric multidimensional scaling (NMDS) analysis (**a**) of the total and active PAM and FLM communities incubated with ^13^C- or ^12^C-POM at 0.1, 20, and 40 MPa, based on Bray–Curtis similarity matrix at OTU level. PA-t and PA-a represent total PA and active PA, respectively. Linear discriminant analysis (LDA) (**b**) identified the different indicator microbial groups between 0.1, 20, and 40 MPa, with LDA scores of 5.0.

NMDS and ANOSIM identified four significantly different (*p* < 0.05, Table S5) clusters: the total and active PAM and FLM communities at 0.1 MPa (Ⅰ), 20 MPa (Ⅱ), and 40 MPa (Ⅲ), and the total PAM and FLM in ISW (Ⅳ) (Figure 2a). This result suggests that the microbial communities were significantly influenced by hydrostatic pressure (*p* < 0.05). However, no significant differences (*p* > 0.05, Table S5) were observed between the PAM and FLM communities at all pressures (Figure 2a).

Linear discriminant analysis (LDA) (Figure 2b) indicated the significantly different indicator microbial groups under different pressures. Overall, a total of 13, 12, and 7 indicative phylotypes were detected at 0.1, 20, and 40 MPa, respectively, suggesting that high pressure significantly affected the microbial community composition and decreased the number of bioindicators. For instance, OTU156 (no-rank *Gracilibacter*) was obviously enriched at 0.1 MPa, whereas OTU117 (*Pseudoalteromonas*) and OTU395 (*Tenacibaculum*) were predominant at 20 and 40 MPa, respectively.

### 3.2. Taxonomic Compositions of the PAM and FLM Communities

**Figure 3:**
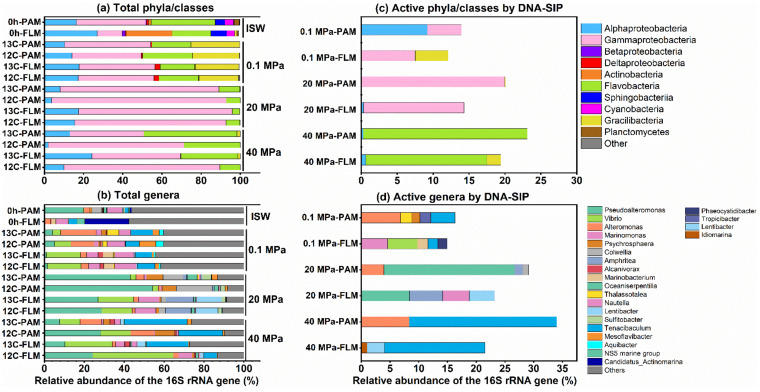
The most abundant total and active PAM and FLM classes and genera (>1% of the relative abundance) for POC utilization at 0.1, 20, and 40 MPa, respectively. (**a**,**b**) The total PAM and FLM communities at phylum/class and genus level, respectively. (**c**,**d**) Active PAM and FLM taxa identified by DNA-SIP at phylum/class and genus level, respectively.

Taxonomic compositions of the total and active PAM and FLM communities and their relative abundances at ISW and different pressures are presented in Figure 3. Gammaproteobacteria, with relative abundance of 5–81%, were the most dominant lineages in the total communities at all the pressures, followed by Bacteroidetes (4–48%), Alphaproteobacteria (1–27%), Gracilibacteria (1–25%), Actinobacteria (2–24%), Cyanobacteria (0-4%), Deltaproteobacteria (1–3%), Planctomycetes (1–3%), and Betaproteobacteria (0–2%) (Figure 3a).

Gammaproteobacteria in the total PAM and FLM communities dramatically increased with pressure (20 MPa) and then sharply decreased at 40 MPa. In contrast, Bacteroidetes reached to the maximum values at 40 MPa (Figure 3a). In addition, Alphaproteobacteria decreased with pressure, and the maximum values of Gracilibacteria only occurred at 0.1 MPa and then disappeared with pressures (Figure 3a). Moreover, Actinobacteria, Cyanobacteria, Planctomycetes, and Betaproteobacteria only presented in ISW (Figure 3a).

Several important genera such as *Alteromonas*, *Vibrio*, *Pseudoalteromonas*, *Thalassotalea*, *Psychrophaera*, and *Colwellia* dominated the Gammaproteobacteria. *Alteromonas* and *Thalassotalea* tended to decrease with pressure, with the average relative abundance of 2–15% and 1–4%, respectively, whereas *Pseudoalteromonas* (1–49%), *Vibrio* (3–32%), and *Psychrosphaera* (1–8%) increased with pressure (Figure 3b). Two key taxa, *Tenacibaculum* and *Mesoflavibacter*, were the predominant members among Bacteroidetes. *Tenacibaculum* in both PA and FL fractions increased with pressure, with the average proportion of 2–27% (Figure 3b). Moreover, *Nautella* and *Lentibacter* were the most abundant taxa in Alphaproteobacteria. They dramatically decreased with pressure, accounted for 1–10% and 1–12%, respectively (Figure 3b).

DNA-SIP results clearly revealed that members of Gammaproteobacteria, Bacteroidetes, and Alphaproteobacteria were responsible for POM utilization in the active PAM and FLM communities (Figure 3c). As mentioned above, similar variation trends with pressure were observed in Gammaproteobacteria, Bacteroidetes, and Alphaproteobacteria (Figure 3c). At genus level, the active PAM microbial taxa were mainly affiliated with *Alteromonas* (7%) and *Tenacibaculum* (4%); *Pseudoalteromonas* (23%) and *Alteromonas* (4%); and *Tenacibaculum* (26%) and *Alteromonas* (8%) at 0.1, 20, and 40 MPa, respectively (Figure 3d). The active FLM genera included *Vibrio* (5%) and *Marinomonas* (5%); *Pseudoalteromonas* (8%) and *Amphritea* (6%); *Tenacibaculum* (17%) and *Lentibacter* (3%) at 0.1, 20, and 40 MPa, respectively (Figure 3d).

### 3.3. Microbial Preference for PA or FL Lifestyles

**Figure 4:**
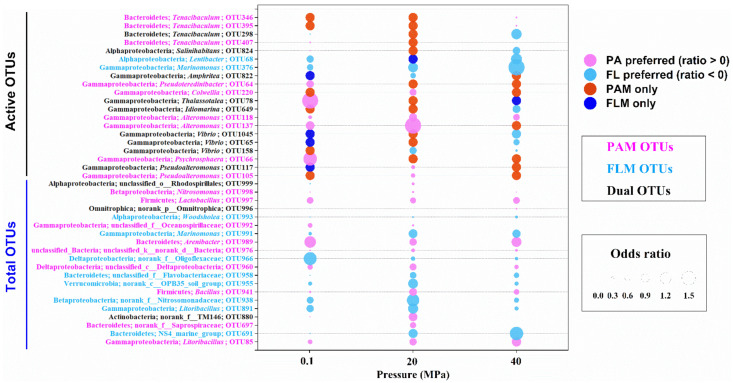
Odds ratio for the total and active OTUs during POM sinking at 0.1, 20, and 40 MPa, respectively. Fuchsia bubbles represent the PA preference with odds ratio > 0, whereas light blue bubbles represent the FL preference with odds ratio < 0. Red and blue bubbles mean only PA and FL lifestyle, respectively. Six scales are shown at the right.

The odds ratio was used to determine the preference of bacteria to the PA or FL lifestyle [54]. Odds ratio > 0 indicates PA preference, whereas odds ratio < 0 represents FL preference. Odds ratios of the total and active bacterial taxa (>1% of relative abundance) at the OTU level and at different pressures are shown in Figure 4.

For top 20 most abundant OTUs of the total microbial communities, the dominant bacterial lineages (9/20) for PA preference comprised Betaproteobacteria (genus *Nitrosomonas*), Gammaproteobacteria (*Litoribacillus* and *Oceanospirillaceae*), Bacteroidetes (*Arenibacter* and Saprospiraceae), and others (Figure 4). By contrast, the predominant lineages (8/20) for FL preference mainly consisted of Alphaproteobacteria (*Woodsholea*), Betaproteobacteria (Nitrosomonadaceae), and Gammaproteobacteria (*Marinomonas* and *Litoribacillus*), etc. However, only three unclassified or no-rank OTUs (OTU999, OTU996, and OTU880) were potentially generalists with PA and FL dual lifestyles with different hydrostatic pressures (Figure 4).

For a total of 20 active OTUs, nearly half of the bacterial lineages (9/20) exhibited a preference for the PA lifestyle at all pressures, mainly including members of Gammaproteobacteria and Bacteroidetes, whereas only two bacterial taxa, *Lentibacter* and *Marinomonas*, showed a preference for the FL lifestyle under all pressures (Figure 4). Most interestingly, a considerable number of active bacterial lineages had PA and FL dual lifestyles at different pressures. For instance, some bacterial lineages such as *Tenacibaculum*, *Salinihabitans*, *Thalassotalea*, *Idiomarina*, and *Vibrio* switched their lifestyles from PA preference at low pressures (0.1 or 20 MPa) to FL preference at high pressure (40 MPa), whereas other microorganisms, including *Amphritea* and *Pseudoalteromonas*, changed from FL preference at low pressure to PA preference at high pressure (Figure 4). Actually, a high proportion of the shared OTUs between the PAM and FLM assemblages were observed, accounting for 63–73% of the total communities and 14–27% of the active communities (Figure S3).

### 3.4. Ecological Networks

**Figure 5:**
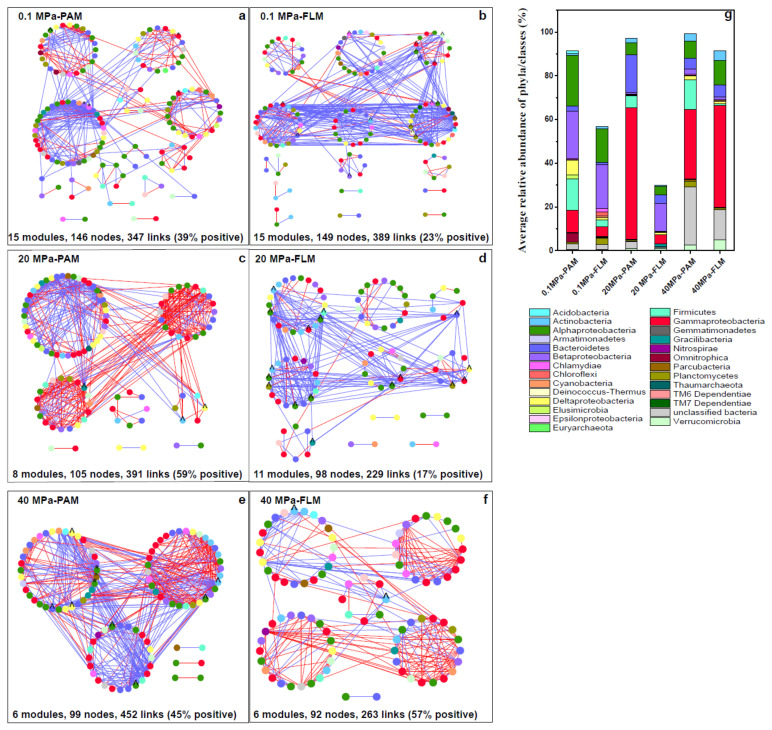
Network analysis of the particle-attached (PAM) and free-living microbial (FLM) communities during POM sinking at 0.1, 20, and 40 MPa, respectively. The networks were constructed using the MENA pipeline as described in Materials and Methods. Each node represents an OTU, and color of the nodes represents different phyla or classes of microorganisms. Each link represents either a positive (red line) or negative (blue line) correlation. Nodes with asterisk label (*) and label ^ represent module hubs and connectors, respectively. (**a**) 0.1 MPa-PAM; (**b**) 0.1 MPa-FLM; (**c**) 20 MPa-PAM; (**d**) 20 MPa-FLM; (**e**) 40 MPa-PAM; (**f**) 40 MPa-FLM. (**g**) Average relative abundance of the PA or FL microbial taxonomic groups at phylum or class level under the three different pressures in the constructed networks.

A total of six networks were constructed for the PAM and FLM communities at three different pressures (Figure 5). The constructed networks consisted of 274 OTUs, with nodes representing OTUs and links suggesting correlations (positive or negative) between OTUs (Figure 5 and Table 1). The overall topology indexes suggest that the PAM and FLM networks exhibited scale-free and small-world features, as indicated by higher average clustering coefficient (avgCC), average geodesic distance (GD), and modularity, which were significantly different from the respective randomized networks (*p* < 0.01, Table 1). Most importantly, PAM networks showed more complexity than FLM networks, with a higher average connectivity (avgK), higher avgCC, and smaller GD at high pressures (i.e., 20 and 40 MPa) (Table 1).

For PAM networks, a total of 29 modules were generated, and the 350 nodes were affiliated with 21 phyla, mainly including Proteobacteria (44.1–66.6%), Bacteriodetes (2.3–17.3%), and Firmicutes (5.5–14.3%), with Proteobacteria being the most abundant phylum (Table 1 and Figure 5g). The number of nodes, modules, and modularity decreased with pressure, whereas the number of links increased with pressure (Table 1). Meanwhile, the higher avgK, higher avgCC, and smaller GD were observed at elevated pressure (Table 1), suggesting that increasing HP enhanced network complexity and microbial interactions for the PAM networks. Overall, the PAM networks consisted of highly connected OTUs forming structured modules.

The FLM networks were slightly larger (32 modules), and the 339 nodes in the FLM networks were mainly associated with Proteobacteria (21.8–60.2%), Bacteriodetes (1.1–5.4%), and Verrucomicrobia (0.6–4.9%) (Table 1 and Figure 5g). Similar to the PAM networks, the number of nodes and modules decreased steadily with pressure. However, avgK, avgCC, modularity, and number of links in the FLM networks decreased from 0.1 to 20 MPa, and then increased at 40 MPa, with the smallest GD (3.52) at 40 MPa (Table 1).

**Figure 6:**
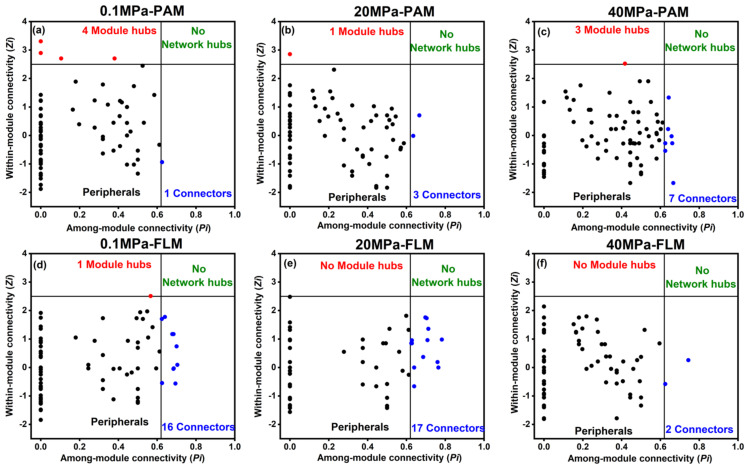
ZP−plot showing putative keystone species within the PAM and FLM networks during POM sinking at 0.1, 20, and 40 MPa, respectively. Each dot represents an OTU from the six networks selected for detailed module analysis (Figure 5). The module hubs (Zi > 2.5) and connectors (Pi > 0.62) are shown as red and blue dots, respectively. Detailed taxonomic information for module hubs and connectors is listed in Table S6. (**a**) 0.1 MPa−PAM; (**b**) 20 MPa−PAM; (**c**) 40 MPa−PAM; (**d**) 0.1 MPa−FLM; (**e**) 20 MPa−FLM; (**f**) 40 MPa−FLM.

Module hubs and connectors are considered keystone species and play an essential role in structuring ecological networks [27,56,63]. Module hubs are responsible for structuring the networks and maintaining network stability, whereas connectors are mainly “communicators” in information processing and transfer in the networks [26,27,28]. A total of nine module hubs and 46 connectors were identified in the PAM and FLM networks in our study (Figure 6 and Table S6). However, no network hubs were found for any of the constructed networks. The PAM networks had eight module hubs, whereas only one module hub was found in the FLM networks. In contrast, the number of connectors identified in the FLM networks (35 connectors) was much more than that in the PAM networks (11 connectors). Therefore, there were more module hubs in the PAM networks and more connectors in the FLM networks, suggesting the different ecological roles of the PAM and FLM communities during POM sinking (Figure 6 and Table S6).

The eight module hubs identified in the PAM networks were assigned to Proteobacteria (α-, β-, and γ-proteobacteria), Chlamydiae, Cyanobacteria, and Parcubacteria. On the other hand, only one module hub, member of Acidobacteria, was detected in the 0.1 MPa-FLM network (Figure 6 and Table S6).

Connectors were detected in all PAM and FLM networks, but the taxonomic compositions of connectors were rather different between the PAM and FLM networks. For instance, the 11 connectors identified in the three PAM networks consisted of members of Proteobacteria (including α- and δ-proteobacteria), Actinobacteria, Bacteroidetes, and Gracilibacteria (Figure 6 and Table S6). On the other hand, 35 connectors in the three FLM networks originated from a variety of taxonomic groups: 16 belonged to Proteobacteria (primarily α-, β-, γ-, and δ-proteobacteria), 5 belonged to Actinobacteria, and others to Bacteroidetes, Chlamydiae, Deinococcus-Thermus, Firmicutes, Gracilibacteria, Parcubacteria, Planctomycetes, and Verrucomicrobia (Figure 6 and Table S6).

## 4. Discussion

### 4.1. Microbial Succession and Lifestyle Preferences during POM Sinking

Our results suggest a significant decrease in microbial diversity under HHP and LT conditions (Figure 1). One possible explanation is that the metabolic activity and cellular processes of microbial communities seem to be inhibited by HHP and LT; therefore, a large number of microbial cells may die or tend to be dormant under unfavorable environments [30,32,64,65]. Similar variation trends were observed in microbial communities of pressure-induced sediment samples [64] and during sinking particles degradation [30].

On the other hand, the Shannon diversity index was higher in the FLM communities than in PAM communities, especially at 0.1 and 20 MPa (Figure 1). It is well known that carbon- and nutrient-rich diatom particles serve as hotspots for microbial colonization and utilization [4,66]. However, only a minority of PA bacterial taxa (so-called r-strategists) [8,26] could rapidly attach to particles and further decompose organic particles due to competing for limited nutrients [9]; therefore, the growth of the other PAM species may be inhibited, or the species may even die. These factors may ultimately result in lower microbial diversity of PAM communities [9]. In contrast, the labile DOM plume, released from decomposition of the sinking POM aggregates, may promote the growth and diversity of FLM communities (i.e., K-strategists) adapted to the oligotrophic environments [4,8,66]. Similar findings were reported in the water column of the South China Sea [10,67] and the New Britain Trench [9].

Hydrostatic pressure is a key environmental factor in shaping microbial community structure during POM sinking [20,29,30,36]. NMDS and LDA analyses showed that the microbial communities formed different clusters according to hydrostatic pressure, irrespective of microbial lifestyle (Figure 2). ANOSIM (analysis of similarity) further demonstrated that there were significant differences in microbial communities at different pressures (Table S5). These findings are consistent with findings from previous pressure incubation experiments with surface seawaters [36], sediments [64], and sinking POM [20,29].

Piezophiles are defined as organisms that have higher growth rates at high hydrostatic pressure (HHP) than at atmospheric pressure (0.1 MPa), whereas piezotolerant have higher growth rates at atmospheric pressure than at high pressure [31,32]. In this study, we observed that members of Bacteroidetes (e.g., *Tenacibaculum*) were the most abundant and active lineages for POM utilization at high pressure (40 MPa) (Figure 3), which implied that members of Bacteroidetes were cultivable piezophilic microorganisms. In contrast, members of Gammaproteobacteria, Alphaproteobacteria, and Gracilibacteria decreased with pressure (Figure 3), suggesting that these microorganisms may be identified as piezotolerant. A relevant study found that Bacteroidetes were piezophiles in high-pressure enrichment cultures from 1.5 to 2.4 km-deep coal-bearing sediments by applying 16S rRNA gene sequencing [68]. Additionally, the recent advances revealed that the genomes of deep-sea Bacteroidetes contained genes encoding TmaT-like and MpTmm-like proteins, which transported and oxidized trimethylamine (TMA) into trimethylamine N-oxide (TMAO), and the presence of TMAO could improve tolerance of HHP stress, implying a common HHP tolerance strategy adopted by Bacteroidetes [69].

How could surface water microorganisms, including piezosensitive and piezotolerant microbes, adapt to HHP and LT? Mestre and coworkers observed that the most abundant microorganisms in the bathyplegic zones of the Atlantic, Pacific, and Indian oceans were also present in surface waters, and they hypothesized that sinking particles may act as important vectors that transfer particle-attached surface microbes into the deep ocean and connect surface communities to deep-sea microbes, further determining to some extent the composition of deep ocean communities [5].

DNA-SIP results reveal that members of Gammaproteobacteria, Alphaproteobacteria, and Bacteroidetes were the potential key players during POM sinking, which agreed well with earlier studies [20,70,71,72]. Many studies also found that the deep-water microbial communities of the ECS were dominated by Alphaproteobacteria, Gammaproteobacteria, Bacteroidetes, etc. [73,74,75], which was in accordance with our study.

Several predominant species within the Gammaproteobacteria, including *Alteromonas*, *Pseudoalteromonas*, *Vibrio*, and *Marinomonas*, had the special capabilities for utilization of diatom-derived POM (Figure 3). As important colonists of particles, *Alteromonas* and *Marinomonas* played major roles in the utilization of diatom-derived organic matter and marine DOM [76,77,78,79]. *Pseudoalteromonas* could secrete a large number of ectoenzymes to degrade diatom detritus and high molecular weight DOM [72,80,81]. In addition, *Vibrio*, known as a type species of particle-associated bacterial communities, enables the formation of biofilms and produces extracellular enzymes for biopolymer degradation in marine environments [66,82,83,84]. A large number of accumulating findings also demonstrated that Bacteroidetes are well known to be proficient in the utilization of complex organic matter in the deep waters of the ECS [74] and other aquatic environments [85,86,87,88].

It is important to note that a large number of active bacterial taxa exhibited different preferences for PA or FL lifestyles under HHP and LT conditions (Figure 4 and Figure S4). This result suggests that these bacterial lineages were potentially generalists with dual lifestyles for versatile metabolic strategies [10,22,67,89] and that the lifestyle preferences were clearly affected by HHP and LT. For instance, several active POM degraders such as *Vibrio*, *Tenacibaculum*, *Salinihabitans*, *Thalassotalea*, and *Idiomarina* changed from PA preference to FL preference at HHP and LT; however, *Pseudoalteromonas* and *Amphritea* varied from FL preference to PA preference at HHP and LT [11,66,67].

On the other hand, almost half of the bacterial lineages that showed PA preference could be attributed to their specific abilities for adhesion to particles for nutrient acquisition and degradation of organic matter [11,17,90,91]. In contrast, two bacterial taxa, such as *Woodsholea* and *Lentibacter* in Alphaproteobacteria, exhibited FL preference, likely due to their smaller cell size, streamlined genome, and lower carbohydrate hydrolysis activities [7,24].

### 4.2. Microbial Network Interactions during POM Sinking

HHP and LT are important environmental factors in shaping the structure and complexity of microbial networks [64]. We found that the PAM and FLM networks became more complex with POM sinking, as indicated by their increased connectivity (avgK), tightest clusters (avgCC), and shortest GD with increasing pressure and decreasing temperature. In addition, the PAM networks were observed to be more complex than FLM networks at HHP and LT (Table 1 and Figure 5). One explanation is that HHP will change the equilibrium towards the state that occupies the smallest biovolume [32]; therefore, the distance between any two nodes in a network was shortened, and then the network interactions tended to be enhanced under HHP and LT. In addition, more close interactions were observed in the PAM networks than in the FLM networks, suggesting that a relatively limited space in particles is a more advantageous for interactions within the PAM communities [25].

The stability and robustness of an ecosystem depended on microbial interactions, and lower connectivity and higher modularity in a microbial community resulted in higher functional stability of the ecosystem [56]. In our study, both PAM and FLM networks exhibited higher average connectivity with HHP and LT (Table 1), suggesting that HHP and LT enhanced the microbial interactions but led to greater network vulnerability of the networks. Therefore, the PAM networks tended to be more vulnerable at HHP and LT due to higher connectivity, lower modularity, and lower diversity. On the other hand, the FLM networks had lower connectivity (avgK), higher modularity, and higher alpha-diversity, which may result in greater resilience or stability than the PAM networks [28], particularly at HHP and LT (Table 1 and Figure 1).

The divergent differences in topological features of the PAM and FLM networks affected by HHP and LT could be attributed to microbial trophic strategies and interaction mode [8]. For one, microbes colonized on particles were closer to each other than free-living microorganisms [7,92]; accordingly, more efficient metabolic interactions were likely to occur in the PAM networks [8,11,27,66]. For the other, the PAM networks had more negative associations (55%), while the FLM networks had more positive links (57%) at 40 MPa and 4 °C (Figure 5 and Table 1), indicating that drastic amensalism, predation, and competition (i.e., negative associations) occurred among microbes in the PAM networks, whereas better cooperative behaviors (e.g., cross-feeding, co-aggregation in biofilms, co-colonization, and niche overlap) appeared within the FLM networks [25,27,78,93]. Thus, distinct microbial interactions between PAM and FLM networks may intensify this difference. Together, our results further demonstrate the different ecological functions and niches mediated by the PAM and FLM communities during POM sinking.

Our results further reveal that members of the PAM assemblages constituted most of the network module hubs, while members of the FLM communities served as most network connectors (Figure 6 and Table S6). For example, OTU492 and OTU850 belonged to the family Parachlamydiaceae (phylum Chlamydiae) but played different topological roles as module hub and connector in PAM and FLM networks at 0.1 MPa, respectively (Table S6). A recent study demonstrated that Chlamydiae played a crucial role in plant–bacteria ecological networks and were in connection with Cyclobalanopsis multinervis [94]. Although OTU862 (genus *Pseudogulbenkiania*) and OTU222 (OM43 clade) were both classified to class Betaproteobacteria, OTU862 played an important role as a module hub in the 0.1 MPa PAM network, whereas OTU222 acted as a connector in the 0.1 MPa FLM network (Table S6). The OM43 clade is a methylotroph that can use methanol and formaldehyde as carbon and energy sources [95,96] and is commonly found in coastal ecosystems with low abundance [97]. Members of Betaproteobacteria were also putative keystone taxa (both module hubs and connectors) in the rhizosphere networks [63] and Taihu Lake [28]. Moreover, two gammaproteobacterial taxa (*Alteromonas* and *Marinomonas*) were identified as module hubs in a PAM network. However, six gammaproteobacterial taxa (Alteromonadales, Oceanospirillaceae, *Aquicella*, etc.) were classified as connectors in all FLM networks (Table S6). The gammaproteobacterial species were only identified as module hubs in the rhizosphere network [63]. These results indicate that the PAM assemblages played a more important role in structuring the networks and maintaining network stability, while the FLM taxa were mainly “communicators” in information processing and transfer in the networks.

On the other hand, OTU1034 and OTU104 were both assigned to the phylum Gracilibacteria, which all acted as connectors in PAM and FLM networks at 20 MPa, respectively (Table S6). Relevant reports revealed that archaeal OTUs formed a consortium with Gracilibacteria, which likely involved in nitrogen and methanogen cycles in the deep layers of the lake [98,99]. Seven Actinobacterial microorganisms, including Microbacteriaceae, *Rhodococcus*, *Nocardia*, *Gaiella*, Candidatus Aquiluna, *Iamia*, and *Nocardioides*, were all classified as connectors in the PAM and FLM networks (Table S6). Actinobacteria play important roles in organic matter decomposition and production of secondary metabolites with diverse physiological functions [100]. Additionally, members of Actinobacteria were detected as module hubs and connectors in the rhizosphere networks [63] and during phytoplankton bloom [27], respectively.

Surprisingly, most of the identified module hubs (44.4%) and connectors (50.0%) were unclassified at the genus level and of low abundances (Figure 6 and Table S6), suggesting that taxa of the rare biosphere may play more important roles in maintaining network structures of PAM and FLM communities in the ocean. This finding was similar to that observed in a freshwater lake [27] and soil microbial communities [63,101].

## 5. Conclusions

By combining laboratory simulation of particle sinking in the ocean with DNA-SIP and 16S rRNA gene sequencing, we were able to piece together the community successions and network interactions of the PAM and FLM under different pressures and temperatures during POM sinking. HHP and LT significantly decreased microbial diversity, and higher diversity was observed in the FLM communities than PAM communities. HHP and LT were critical driving forces in shaping microbial community structure, which was largely dominated by members of Bacteroidetes at 40 MPa. A large number of active bacterial taxa possessed PA and FL dual lifestyles and were able to change their lifestyle preference with HHP and LT, implying versatile metabolic strategies. HHP and LT enhanced the networks interaction and resulted in more vulnerability of PAM networks and more stability of FLM networks. Most interestingly, the PAM communities mainly occupied the module hubs of the network, whereas the FLM communities served as most of connectors, suggesting different ecological roles of the PAM and FLM communities during POM sinking. Our findings provide further ecological insights into how HHP and LT affected the community successions and ecological networks of the PAM and FLM during POM sinking and their implications for marine carbon cycling.

## Figures and Tables

**Table 1:** The topological properties of the co-occurrence networks for the PAM and FLM communities at 0.1, 20, and 40 MPa.

0	Network Features	0.1 MPa-PAM (Threshold Value = 0.94)	20 MPa-PAM (0.91)	40 MPa-PAM (0.91)	0.1 MPa-FLM (0.95)	20 MPa-FLM (0.93)	40 MPa-FLM (0.93)
**Empirical networks**	Total OTUs	289	166	181	297	196	187
	Total nodes	146	105	99	149	98	92
	Total links	347	391	452	389	229	263
	Total positive links	136	230	204	88	40	151
	Total negative links	211	161	248	301	189	112
	Total modules	15	8	6	15	11	6
	Modularity	0.60	0.52	0.35	0.49	0.40	0.60
	Average connectivity (avgK)	4.75	7.45	9.13	5.22	4.67	5.72
	Average clustering coefficient (avgCC)	0.27	0.32	0.37	0.17	0.12	0.28
	Average geodesic distance (GD)	4.24	3.46	2.57	4.23	5.56	3.52
**Random networks**	Average clustering coefficient (avgCC)	0.06 ± 0.01	0.12 ± 0.01	0.16 ± 0.02	0.08 ± 0.01	0.11 ± 0.02	0.08 ± 0.01
	Average geodesic distance (GD)	3.24 ± 0.05	2.64 ± 0.04	2.40 ± 0.03	3.11 ± 0.05	3.04 ± 0.08	2.82 ± 0.04
	Modularity	0.41 ± 0.01	0.28 ± 0.01	0.24 ± 0.01	0.37 ± 0.01	0.37 ± 0.01	0.34 ± 0.01

## Data Availability

All raw sequence datasets of 16S rRNA genes from this study have been deposited into the NCBI Sequence Read Achieve (SRA) database with the accession no. PRJNA597244.

## Supplementary Materials

The following are available online at https://www.mdpi.com/article/10.3390/microorganisms10030639/s1, Figure S1: Location of the seawater sampling site in the East China Sea (the red dot), Figure S2: High-pressure incubation equipment. (a) Schematic of four 710 mL incubation pouches; (b) schematic of the pressure vessels; (c) schematic of a hand-operated pump, Figure S3: The quantitative distribution of the 16S rRNA gene in the 3rd–14th DNA fractions across different buoyant density gradients of the PAM and FLM assemblages incubated with ^13^C- or ^12^C-POC at 0.1, 20, and 40 MPa. (a) 0.1 MPa-PAM; (b) 0.1 MPa-FLM; (c) 20 MPa-PAM; (d) 20 MPa-FLM; (e) 40 MPa-PAM; (f) 40 MPa-FLM, Figure S4: Venn diagrams showing the number and percentage of shared and unique OTUs in the ISW, total, and active PAM and FLM assemblages incubated at 0.1, 20, and 40 MPa. ISW represents the East China Sea in situ surface water. Table S1: The physical and chemical parameters of the surface water collected from the East China Sea.; Table S2: The 16S rRNA gene sequencing data of PAM and FLM at 0.1, 20, and 40 MPa, respectively; Table S3: Diversity indices of the total and active PA and FL microbial 16S rRNA gene at 0.1, 20, and 40 MPa, respectively; Table S4: The P values and significance of alpha diversity indices and OTUs number between different comparisons in the total and active microbial communities; Table S5: Analysis of similarity (ANOSIM) between the PAM and FLM assemblages before and after incubation at 0.1, 20, and 40 MPa, respectively; Table S6: phylogenetic classification and average relative abundance of module hubs and connectors for the PAM and FLM communities at 0.1, 20, and 40 MPa. File S1. The sequences of plasmid DNA to generate standard curve of qPCR.
