# Supplementary material for: Microbial Community Structure and Ecological Networks during Simulation of Diatom Sinking
Source: Microorganisms. 2022 Mar 17;10(3):639. doi: 10.3390/microorganisms10030639 (PMC8949005; doi:10.3390/microorganisms10030639)
Supplement: Supplementary file 1 [file microorganisms-10-00639-s001.zip › microorganisms-1630895- Supplementary.pdf]

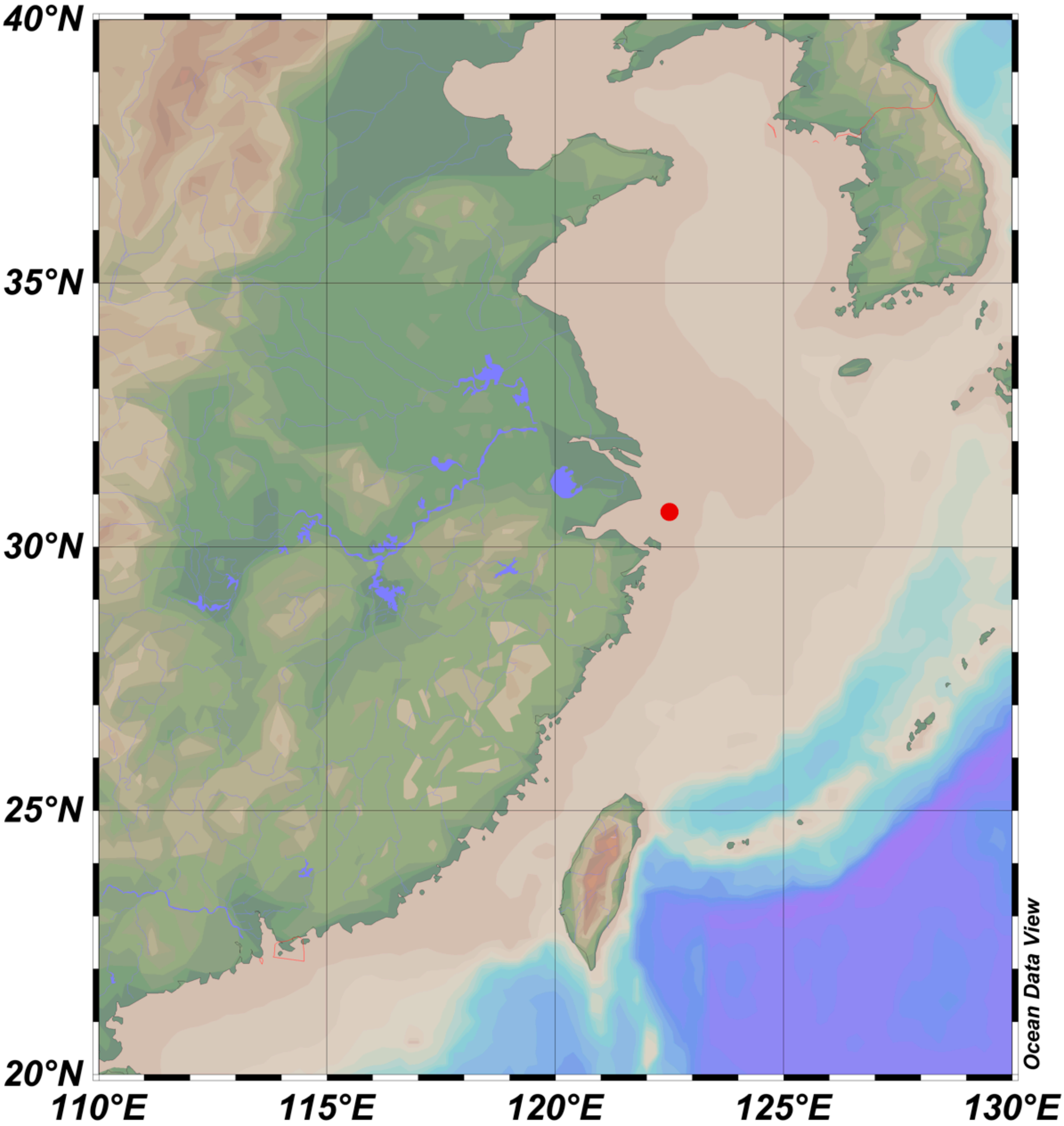

Figure S1. Location of the seawater sampling site in the East China Sea (the red dot).

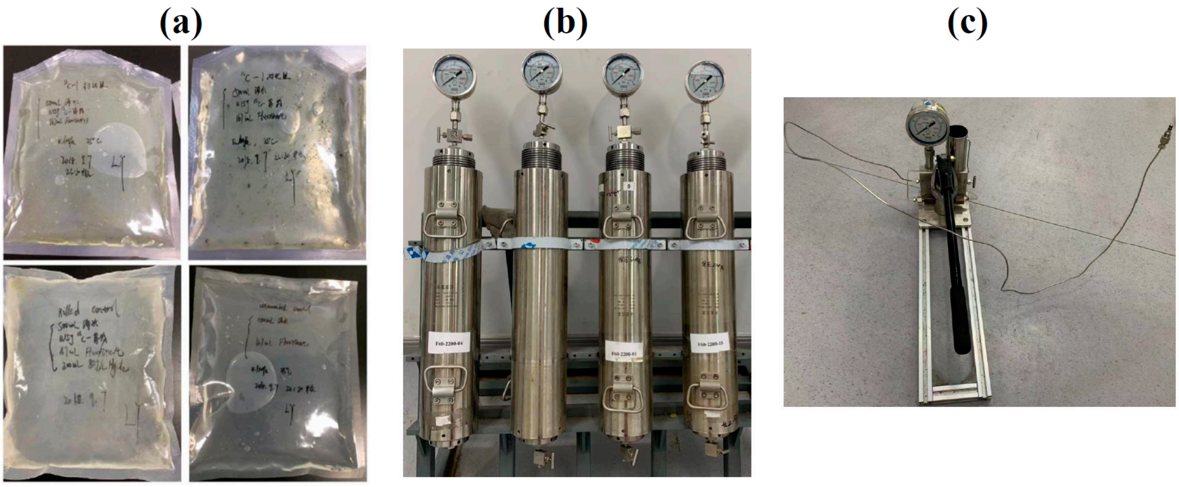

Figure S2. High-pressure incubation equipments. (**a**) Schematic of four 710-mL incubation pouches; (**b**) schematic of the pressure vessels; (**c**) schematic of a hand-operated pump.

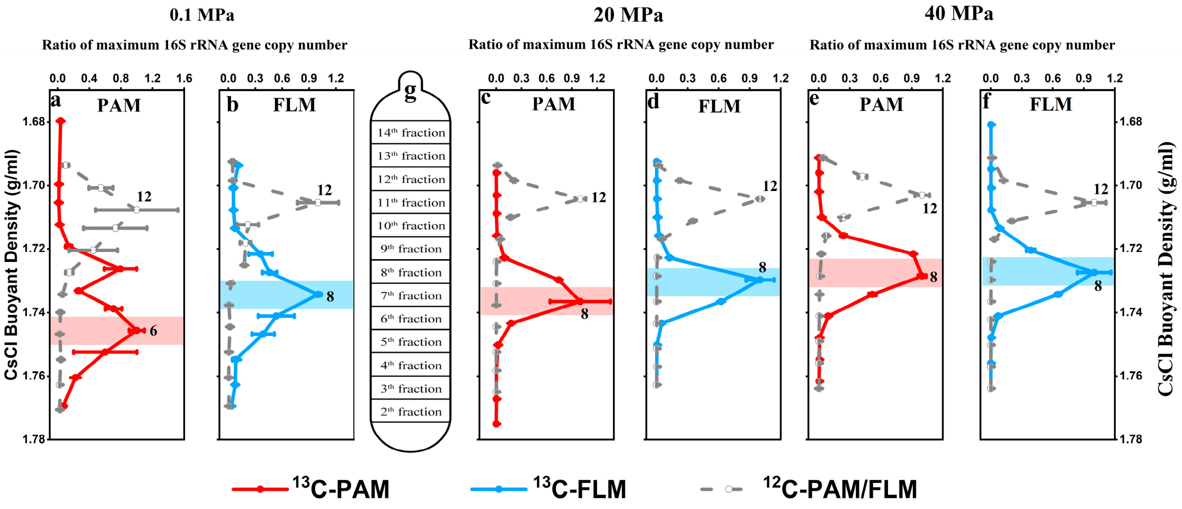

Figure S3. The quantitative distribution of the 16S rRNA gene in the 3th-14th DNA fractions across different buoyant density gradients of the PAM and FLM assemblages incubated with ^{13}C- or ^{12}C-POC at 0.1, 20 and 40 MPa. (**a**) 0.1 MPa-PAM; (**b**) 0.1 MPa-FLM; (**c**) 20 MPa-PAM; (**d**) 20 MPa-FLM; (**e**) 40 MPa-PAM; (**f**) 40 MPa-FLM.

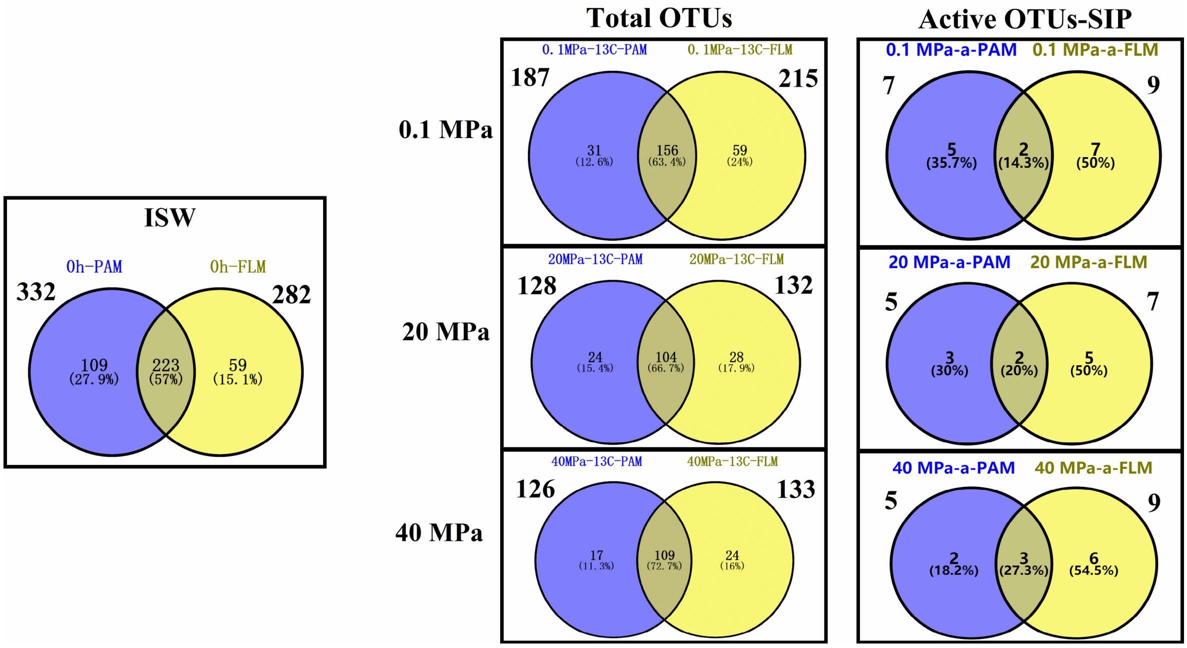

Figure S4. Venn diagrams showing the number and percentage of shared and unique OTUs in the ISW, total, and active PAM and FLM assemblages incubated at 0.1, 20, and 40 MPa. ISW represents the East China Sea in-situ surface water.

Table S1. The physical and chemical parameters of the surface water collected from the East China Sea.

Depth (m)	Temperature (°C)	Salintiy (‰)	Dissolved oxygen (ml L^{-1})	POC concentration(mg L^{-1})	DOC concentration(mg L^{-1})
15	25	33	0.08	0.14	1.02

Table S2. The 16S rRNA gene sequencing data of PAM and FLM at 0.1, 20, and 40 MPa, respectively.

Sample number	Sequence number	Total OTUs	OTUs of PAM	OTUs of FLM	Shared OTUs between PAM and FLM
78	3,615,779	1033	929	835	731

Table S3. Diversity indices of the total and active PA and FL microbial 16S rRNA gene at 0.1, 20, and 40 MPa, respectively.

Sample Name			Sequences	OTUs	Shannon	Chao	Coverage (%)
ISW		PAM	39026	396	2.71	403.75	99.8
		FLM	69244	393	3.81	453.63	99.9
0.1MPa-total	^{13}C-labeled	PAM	66093	259	3.19	306.13	99.9
		FLM	40076	267	3.57	303.35	99.9
	^{12}C-control	PAM	50429	265	3.34	314.04	99.9
		FLM	70471	294	3.64	356.28	99.9
20MPa-total	^{13}C-labeled	PAM	59114	175	2.69	198.66	99.9
		FLM	30024	160	2.67	191.62	99.9
	^{12}C-control	PAM	41372	145	2.10	172.74	99.9
		FLM	69275	190	2.75	231.20	99.9
40MPa-total	^{13}C-labeled	PAM	59342	167	2.89	204.63	99.9
		FLM	44942	162	2.94	198.55	99.9
	^{12}C-control	PAM	29546	112	2.34	130.47	99.9
		FLM	36351	132	2.42	150.82	99.9
0.1MPa-SIP-active	^{13}C-labeled	PAM	35756	202	3.13	226.00	99.9
		FLM	22218	253	3.36	181.96	99.9
	^{12}C-control	PAM	41744	163	3.29	292.23	99.9
		FLM	26979	225	3.61	241.77	99.9
20MPa-SIP-active	^{13}C-labeled	PAM	34219	136	1.91	174.77	99.9
		FLM	38777	146	3.25	384.07	100
	^{12}C-control	PAM	69650	365	2.29	162.84	99.9
		FLM	49472	438	3.24	466.53	99.9
40MPa-fraction	^{13}C-labeled	PAM	42374	133	2.24	167.08	99.9
		FLM	59711	144	2.75	214.07	99.9
	^{12}C-control	PAM	50597	171	2.55	192.05	99.9
		FLM	28458	144	2.41	170.09	99.9

Table S4. The P values and significance of alpha diversity indices and OTUs number between different comparisons in the total and active microbial communities.

Comparisons		Shannon index		Simpsoneven index		Chao1 index		OTUs number	
		P value	Significance	P value	Significance	P value	Significance	P value	Significance
Total-PA	0.1 vs. 20 MPa	0.0001	***	0.00004	***	0.0000003	***	##########	***
	0.1 vs. 40 MPa	0.0007	***	0.24278	NS	0.0000319	***	##########	***

	20 vs. 40 MPa	0.2628	NS	0.00024	***	0.3746099	NS	##########	NS
Total-FL	0.1 vs. 20 MPa	0.0000	***	0.00013	***	0.0001875	***	##########	***
	0.1 vs. 40 MPa	0.0001	***	0.62306	NS	0.0000590	***	##########	***
	20 vs. 40 MPa	0.8378	NS	0.13404	NS	0.1429956	NS	##########	*
Total-PA vs. FL	ISW	0.33185	NS	0.50877	NS	0.423	NS	0.705958901	NS
	0.1 MPa	0.00007	***	0.00041	***	0.268	NS	0.003701512	**
	20 MPa	0.04651	*	0.00003	***	0.135	NS	0.004260844	**
	40 MPa	0.75391	NS	0.46776	NS	0.788	NS	0.324565078	NS
Active-PA	0.1 vs. 20 MPa	0.0009	***	0.01104	*	0.1973044	NS	##########	***
	0.1 vs. 40 MPa	0.0032	**	0.12632	NS	0.0511970	NS	##########	**
	20 vs. 40 MPa	0.1322	NS	0.08799	NS	0.8411168	NS	##########	NS
Active-FL	0.1 vs. 20 MPa	0.0001	***	0.54958	NS	0.0049355	**	##########	***
	0.1 vs. 40 MPa	0.0010	**	0.97744	NS	0.0138336	*	##########	**
	20 vs. 40 MPa	0.0187	*	0.51330	NS	0.1434241	NS	##########	NS
Active-PA vs. FL	0.1 MPa	0.14195	NS	0.56260	NS	0.048	*	0.013106854	*
	20 MPa	0.04016	*	0.07210	NS	0.734	NS	0.329460331	NS
	40 MPa	0.09487	NS	0.44516	NS	0.316	NS	0.302023196	NS

Table S5. Analysis of similarity (ANOSIM) between the PAM and FLM assemblages before and after incubation at 0.1, 20, and 40 MPa, respectively.

	ANOSIM		R value	P value
Comparison 1	Total-PA vs. total-FL	ISW	0.667	0.101
Comparison 2		0.1 MPa	1.000	0.109
Comparison 3		20 MPa	1.000	0.089
Comparison 4		40 MPa	0.482	0.100
Comparison 5	Active PA vs. active-FL	0.1 MPa	0.333	0.277
Comparison 6		20 MPa	0.630	0.100
Comparison 7		40 MPa	0.519	0.100
Comparison 8	Total	ISW vs. 0.1 MPa	0.865	0.001***
Comparison 9		ISW vs. 20 MPa	0.848	0.001***
Comparison 10		ISW vs. 40 MPa	0.878	0.001***
Comparison 11		0.1 MPa vs. 20 MPa	1.000	0.001***
Comparison 12		0.1 MPa vs. 40 MPa	0.948	0.001***
Comparison 13		20 MPa vs. 40 MPa	0.678	0.001***
Comparison 14	Active	ISW vs. 0.1 MPa	0.759	0.002**

Comparison 15	ISW vs. 20 MPa	0.767	0.001***
Comparison 16	ISW vs. 40 MPa	0.763	0.002**
Comparison 17	0.1 MPa vs. 20 MPa	1.000	0.001***
Comparison 18	0.1 MPa vs. 40 MPa	0.898	0.003**
Comparison 19	20 MPa vs. 40 MPa	0.741	0.003**

Table S6. phylogenetic classification and average relative abundance of module hubs and connectors for the PAM and FLM communities at 0.1, 20, and 40 MPa.

Classification of nodesB3:I58	Groups	OTU ID	Zi	Pi	Phylum/Class	Genus	Ave. Rela.Abun. (%)
Module hubs (Zi≥2.5; Pi < 0.62)	0.1MPa-PAM	OTU118	3.31	0.00	Gammaproteobacteria	*Alteromonas*	0.018
		OTU988	2.90	0.00	Gammaproteobacteria	*Marinomonas*	2.158
		OTU862	2.71	0.38	Betaproteobacteria	*Pseudogulbenkiania*	0.014
		OTU492	2.71	0.10	Chlamydiae	unclassified Parachlamydiaceae	0.014
	20MPa-PAM	OTU590	2.86	0.00	Cyanobacteria	norank Cyanobacteria	0.011
	40MPa-PAM	OTU578	2.52	0.42	Alphaproteobacteria	*Tropicimonas*	0.006
		OTU864	2.52	0.42	Parcubacteria	unclassified Parcubacteria	0.011
		OTU979	2.52	0.42	Alphaproteobacteria	unclassified Alphaproteobacteria Incertae Sedis	0.013
	0.1MPa-FLM	OTU930	2.51	0.57	Acidobacteria	*Candidatus_Solibacter*	0.010
Connectors (Zi < 2.5; Pi≥0.62)	0.1MPa-PAM	OTU882	-0.94	0.63	Alphaproteobacteria	norank Mitochondria	0.013
	20MPa-PAM	OTU893	0.71	0.67	Deltaproteobacteria	unclassified Deltaproteobacteria	0.084
		OTU972	0.71	0.67	Actinobacteria	unclassified Microbacteriaceae	0.022
		OTU1034	-0.01	0.63	Gracilibacteria	norank Gracilibacteria	0.033
	40MPa-PAM	OTU993	-1.67	0.67	Alphaproteobacteria	*Woodsholea*	3.103
		OTU977	-0.27	0.66	Alphaproteobacteria	*Roseomonas*	0.390
		OTU945	-0.03	0.66	Deltaproteobacteria	unclassified Deltaproteobacteria	0.019
		OTU516	1.33	0.64	Actinobacteria	*Rhodococcus*	0.008
		OTU98	0.23	0.64	Bacteroidetes	unclassified Flavobacteriaceae	0.029
		OTU897	-0.27	0.63	Alphaproteobacteria	unclassified Rhodospirillaceae	0.010
		OTU942	-0.54	0.63	Deltaproteobacteria	*Bdellovibrio*	0.014
	0.1MPa-FLM	OTU534	0.09	0.70	Planctomycetes	norank S-70	0.008
		OTU850	0.09	0.70	Chlamydiae	unclassified Parachlamydiaceae	0.006
		OTU852	0.09	0.70	Deinococcus-Thermus	*Deinococcus*	0.008

	OTU925	0.09	0.70	Alphaproteobacteria	*Sphingomonas*	0.006
	OTU927	0.09	0.70	Planctomycetes	norank S-70	0.008
	OTU928	0.74	0.70	Chlamydiae	norank Chlamydiaceae	0.006
	OTU493	-0.56	0.69	Actinobacteria	*Nocardia*	0.014
	OTU1030	-0.03	0.69	Gammaproteobacteria	unclassified Alteromonadales	0.011
	OTU1031	1.17	0.68	Gammaproteobacteria	unclassified Oceanospirillaceae	0.032
	OTU200	-0.05	0.68	Verrucomicrobia	norank OPB35 soil group	0.022
	OTU186	1.17	0.68	Bacteroidetes	*Aureispira*	0.008
	OTU89	1.17	0.68	Alphaproteobacteria	unclassified Rhodobacteraceae	0.006
	OTU952	1.17	0.68	Gammaproteobacteria	*Aquicella*	0.006
	OTU18	1.78	0.64	Bacteroidetes	*Pseudofulvibacter*	0.008
	OTU222	-0.54	0.63	Betaproteobacteria	OM43 clade	0.013
	OTU793	1.70	0.62	Parcubacteria	norank Candidatus Giovannonibacteria	0.016
**20MPa-FLM**	OTU835	0.98	0.78	Gammaproteobacteria	SAR92 clade	0.008
	OTU995	0.98	0.78	Alphaproteobacteria	norank SM2D12	0.010
	OTU109	0.00	0.76	Bacteroidetes	norank NS11-12 marine group	0.013
	OTU1001	0.19	0.76	Deltaproteobacteria	norank mle1-27	0.008
	OTU1026	1.36	0.71	Firmicutes	*Atopostipes*	0.006
	OTU497	1.36	0.71	Actinobacteria	*Gaiella*	0.008
	OTU277	0.96	0.71	Gammaproteobacteria	unclassified Gammaproteobacteria	0.014
	OTU302	1.73	0.70	Planctomycetes	*Planctomyces*	0.013
	OTU944	1.76	0.70	Alphaproteobacteria	*Methylobacterium*	0.010
	OTU93	0.37	0.69	Alphaproteobacteria	*Labrenzia*	0.010
	OTU104	0.00	0.64	Gracilibacteria	norank Gracilibacteria	0.025
	OTU501	-0.66	0.64	Gammaproteobacteria	*Stenotrophomonas*	0.014
	OTU174	0.95	0.63	Alphaproteobacteria	unclassified Holosporaceae	0.053
	OTU500	0.85	0.63	Planctomycetes	*Pirellula*	0.008
	OTU833	0.85	0.63	Alphaproteobacteria	*Pseudophaeobacter*	0.010
	OTU943	0.85	0.63	Actinobacteria	*Candidatus Aquiluna*	0.010
	OTU116	0.85	0.63	Alphaproteobacteria	unclassified Rhodobacteraceae	0.014
**40MPa-FLM**	OTU592	0.26	0.74	Actinobacteria	*Iamia*	0.011
	OTU562	-0.58	0.63	Actinobacteria	*Nocardioides*	0.010
